# Supplementary material for: Gene-editing in patient and humanized-mice primary muscle stem cells rescues dysferlin expression in dysferlin-deficient muscular dystrophy
Source: Nat Commun. 2025 Jan 2;16:120. doi: 10.1038/s41467-024-55086-0 (PMC11695731; doi:10.1038/s41467-024-55086-0)
Supplement: Supplementary file 1 — Supplementary Information [file 41467_2024_55086_MOESM1_ESM.pdf]

Supplementary Information

Gene-editing in patient and humanized-mice primary muscle stem cells rescues dysferlin expression in dysferlin-deficient muscular dystrophy

Helena Escobar[1,2,3]*, Silvia di Francescantonio[1,2,3], Julia Smirnova[1], Robin Graf[1,2,3], Stefanie Müthel[1,2,3], Andreas Marg[1,2,3], Alexej Zhogov[1,2,3], Supriya Krishna,[1,2,3,4], Eric Metzler[1,2,3], Mina Petkova[5], Oliver Daumke[1,4], Ralf Kühn[1], Simone Spuler[1,2,3,4]*

[1] Max Delbrück Center for Molecular Medicine in the Helmholtz Association (MDC), 13125 Berlin, Germany

[2] Charité – Universitätsmedizin Berlin, Corporate Member of Freie Universität Berlin and Humboldt-Universität zu Berlin, Charité Campus Buch, 13125 Berlin, Germany

[3] Muscle Research Unit, Experimental and Clinical Research Center (ECRC), a joint cooperation between the Charité – Universitätsmedizin Berlin and the Max Delbrück Center for Molecular Medicine in the Helmholtz Association (MDC), 13125 Berlin, Germany

[4] Department of Biology, Chemistry, and Pharmacy, Freie Universität Berlin, 14195 Berlin, Germany

[5] MyoPax GmbH, 13125 Berlin, Germany

*Correspondence should be addressed to:

Gene editing: Helena Escobar, Ph.D., Muscle Research Unit, Max Delbrück Center for Molecular Medicine in the Helmholtz Association, Robert-Rössle-Str. 10, 13125 Berlin, Germany (e-mail: helena.escobar@mdc-berlin.de; helena.escobar@charite.de; T+49(0)30450540526).

Clinical translation: Simone Spuler, M.D., same address (e-mail: simone.spuler@charite.de; T+49(0)30450540501).

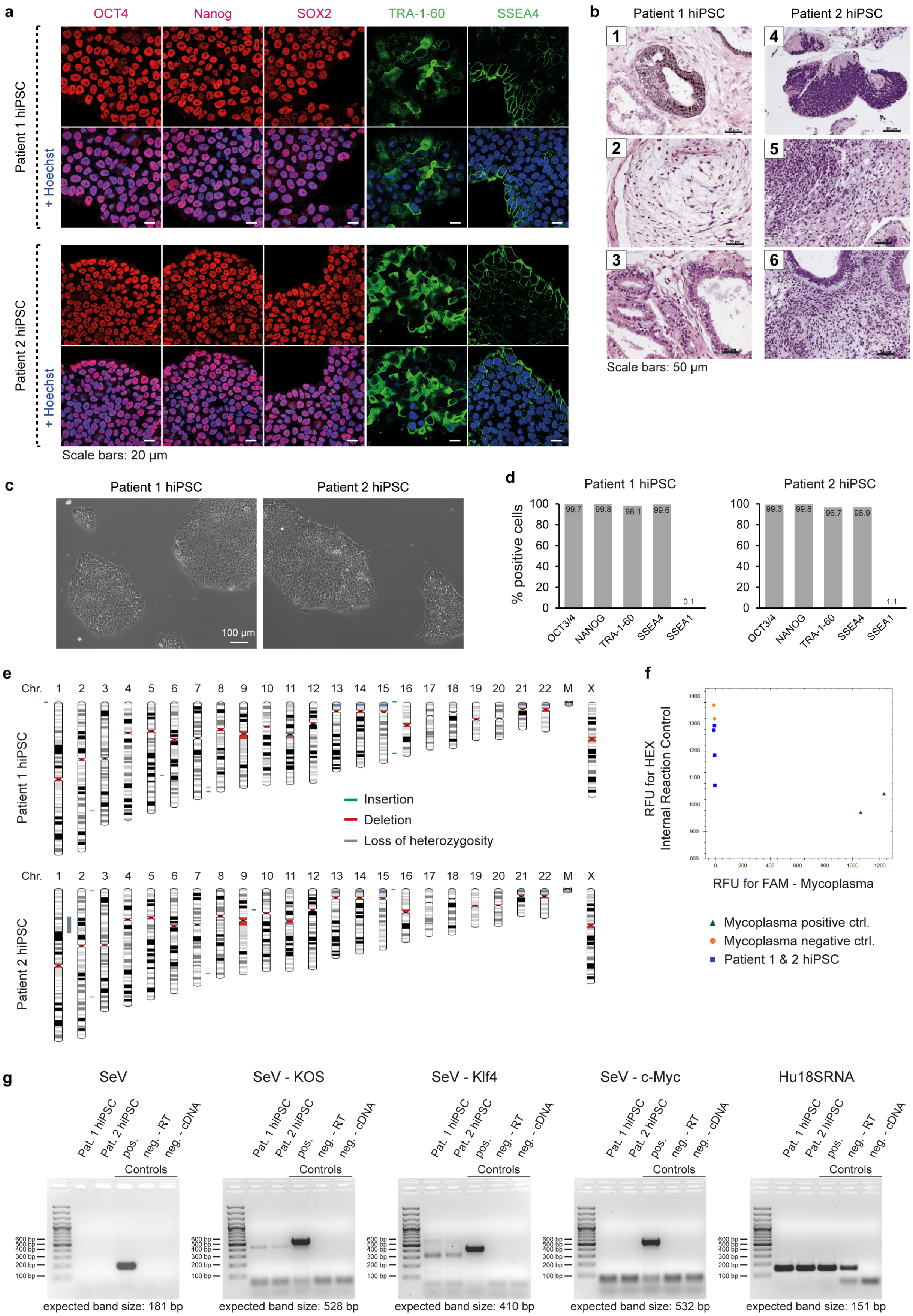

Supplementary Fig. 1: Generation and characterization of patient hiPSC. a hiPSC from two patients immunostained for pluripotency markers. **b** Histopathological analysis of patient hiPSC-derived

teratomas with tissues from the three germ layers: (1) Primitive neurectoderm – beginning pseudorosette formation / Primitive ectoderm – pigment granula; (2) Primitive mesoderm – loose extracellular matrix; (3) Endoderm – cuboidal epithelium and lining cyst-like structure; (4) Neuroectoderm – beginning rosette formation; (5) Mesoderm – immature mesenchyme and connective tissue; (6) Endoderm – isoprismatic to columnar epithelial cells. **c** Bright field microscopy images of hiPSC colonies cultivated in mTeSR1 medium and Matrigel coating. **d** Percentage of cells expressing pluripotency and differentiation markers analyzed by immunostaining and flow cytometry. **e** Virtual karyotype analysis. Regions of gain (duplications) are shown in green, regions of loss (deletions) are shown in red and regions of uniparental disomy (loss of heterozygosity) are shown in grey. Reportable are copy number changes greater than 0.4 Mb compared to the human reference genome and regions of loss of heterozygosity above 3 Mb. **f** Mycoplasma testing by RT-qPCR. X-axis shows relative fluorescence units (RFU) for FAM for Mycoplasma detection. Y-axis shows internal experimental control RFUs for HEX stain. **g** RT-PCR analysis of Sendai virus (SeV) genomes.

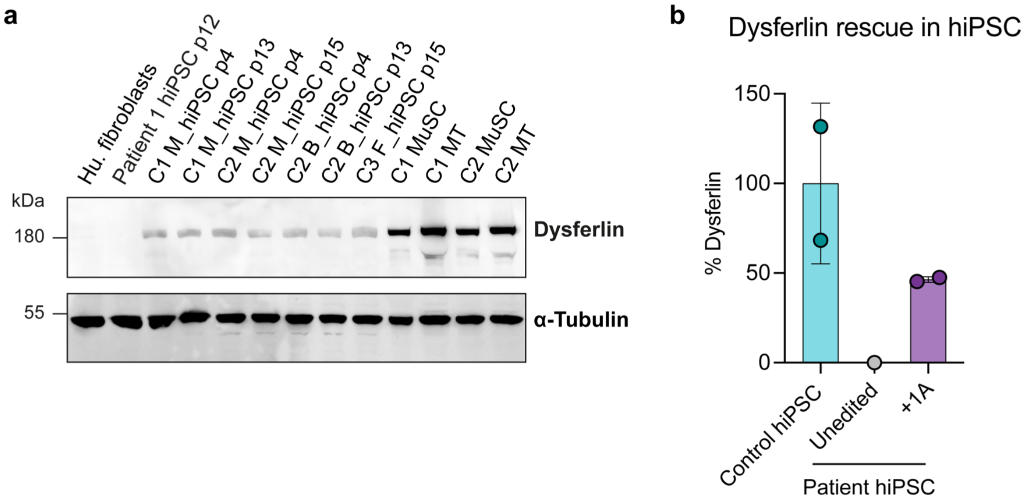

Supplementary Fig. 2: Dysferlin protein is expressed in control hiPSC and is rescued by Cas9-mediated reframing in patient hiPSC. a Western blot analysis of dysferlin protein expression in hiPSC lines derived from MuSC (M_hiPSC), blood (B_hiPSC) or skin fibroblasts (F_hiPSC) from three controls (C1-C3) and from patient 1 at earlier and later passages after reprogramming (p4-p15). Human fibroblasts were used as negative control. Primary MuSC and myotubes (MT) from two controls were used as positive control. α-tubulin was used as loading control (n = 1). **b** Quantification of dysferlin protein rescue in edited patient hiPSC by Western blot densitometry (from Figure 2g). The dysferlin signal is shown relative to alpha-Tubulin and is normalized to the mean of control hiPSC. Source data are provided as a Source Data file.

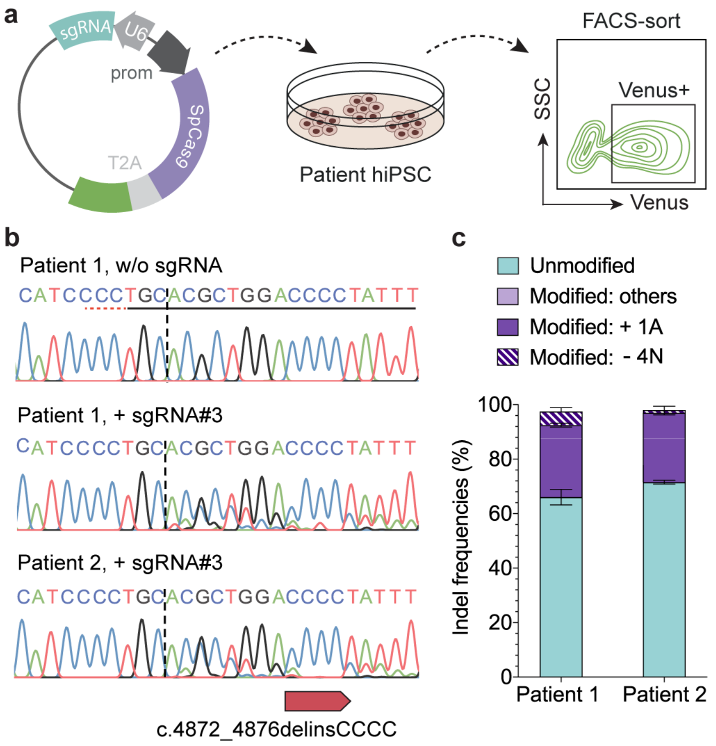

Supplementary Fig. 3: Plasmid-based delivery of wild-type SpCas9 and sgRNA#3 efficiently reframes *DYSF* exon 44 in patient hiPSC. a Schematic overview of experimental workflow. Patient hiPSC were transfected with a plasmid encoding for SpCas9 and a Venus fluorescence reporter, FACS-sorted, and processed for DNA analysis via Sanger sequencing. **b** Sanger sequencing chromatograms of edited (SpCas9, + sgRNA#3) hiPSC from both patients compared to unedited cells (SpCas9, w/o sgRNA). The protospacer and PAM sequences are underlined. The dotted vertical line indicates the expected DSB site. **c** Predicted indel frequencies based on chromatogram deconvolution analysis with ICE (Synthego) (n = 2 repeats per patient hiPSC line; mean ± SD). Source data are provided as a Source Data file.

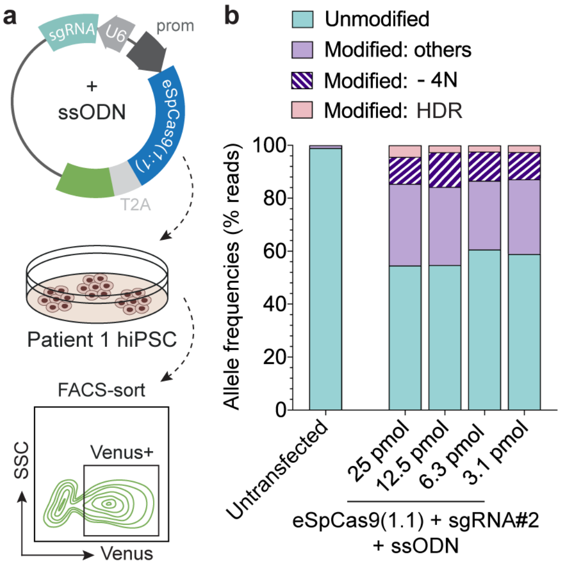

Supplementary Fig. 4: CRISPR-Cas9 mediated HDR in patient hiPSC. **a** Schematic overview of experimental workflow. Patient 1 hiPSC were transfected with a plasmid encoding for eSpCas9(1:1), sgRNA#2 and a Venus reporter, plus different concentrations of a donor template (in the form of a single-stranded oligodeoxynucleotide, ssODN) with the wild-type *DYSF* exon 44 sequence. Venus+ cells were selected by FACS-sorting and processed for DNA analysis via NGS. **b** Allele frequencies determined by NGS (n = 1). Source data are provided as a Source Data file.

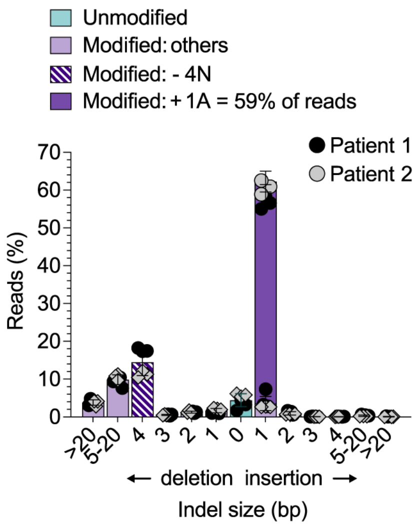

Supplementary Fig. 5: Allele frequencies remain constant throughout cultivation time in edited patient MuSC. Frequency distribution of all indels in MuSC from the two patients at day 9 post-transfection with SpCas9 mRNA and sgRNA#3 (*n* = 3 repeats per patient, mean ± SD). Source data are provided as a Source Data file.

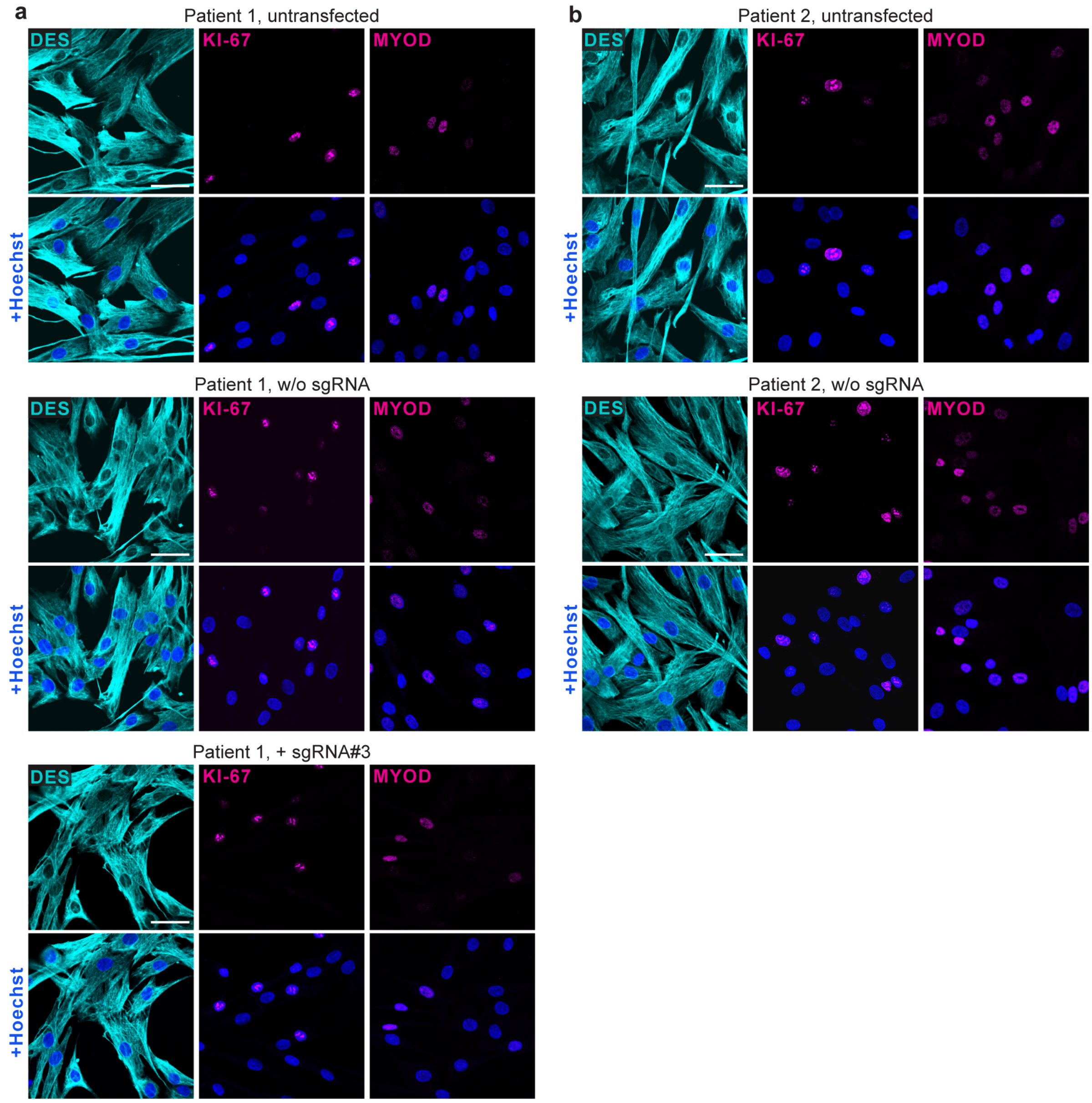

Supplementary Fig. 6: Marker profiling of edited and unedited patient MuSC. MuSC from patient 1 (**a**) and 2 (**b**) were stained for DES, PAX7 and MYOD 4 days after nucleofection with Cas9 mRNA, with or without sgRNA#3. The upper panels show untransfected cells from identical origin that were cultivated in parallel and processed for analysis at the same time. Blue: Hoechst. Scale bar: 50 µm. A quantitative analysis is shown in Figure 4e and corresponding source data are provided as a Source Data file.

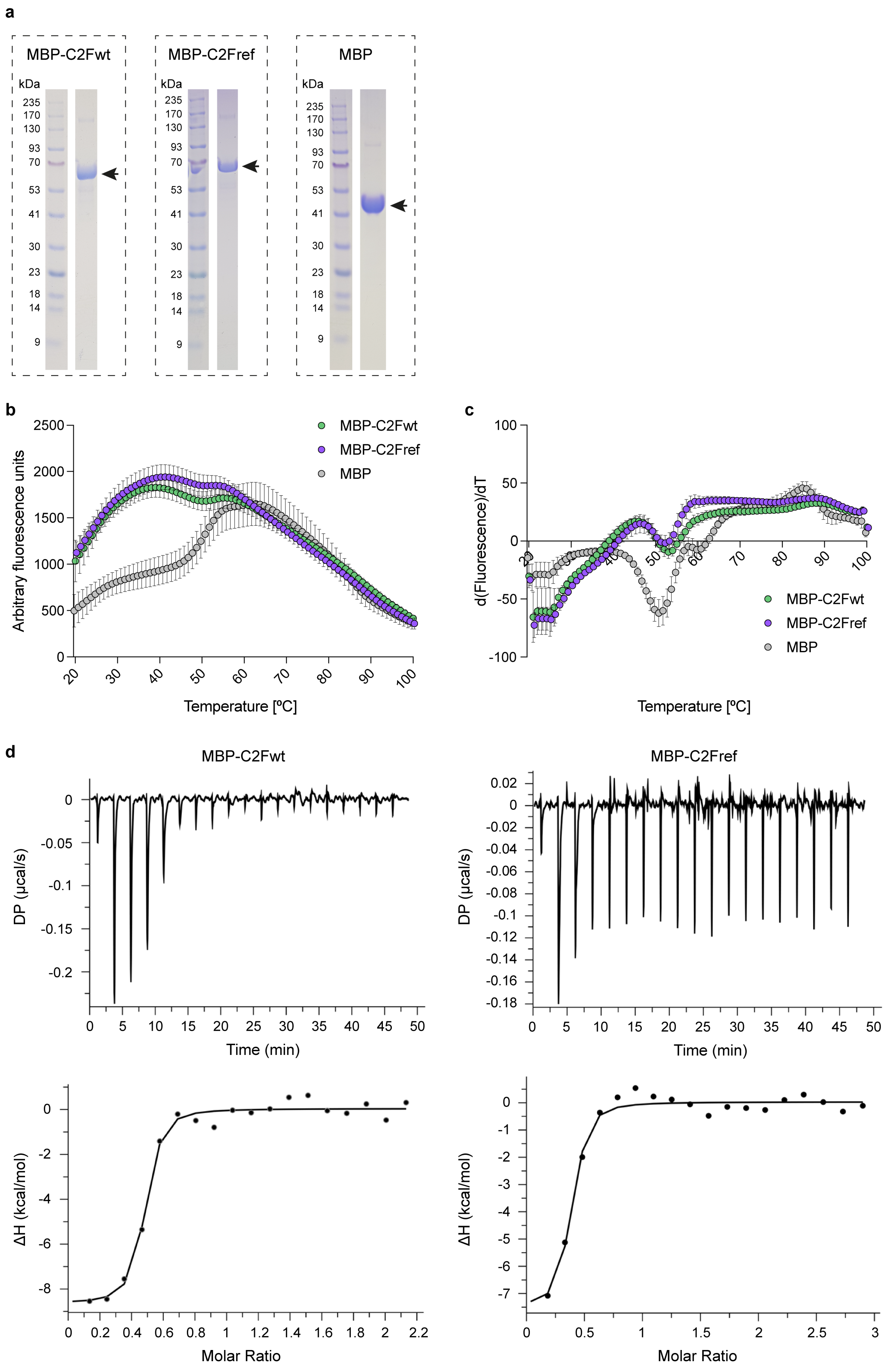
a
MBP-C2Fwt
MBP-C2Fref
MBP
kDa
235
170
130
93
70
53
41
30
23
18
14
9
b
Arbitrary fluorescence units
Temperature [ºC]
MBP-C2Fwt
MBP-C2Fref
MBP
c
d(Fluorescence)/dT
Temperature [ºC]
MBP-C2Fwt
MBP-C2Fref
MBP
d
MBP-C2Fwt
MBP-C2Fref
DP (µcal/s)
Time (min)
ΔH (kcal/mol)
Molar Ratio

Supplementary Fig. 7: Biophysical characterization of the C2F domain. a SDS-PAGE analysis of the proteins used for Thermal Shift and ITC assays. 10 µl of a ~ 10 µM solution of MBP-C2Fwt (left), MBP-C2Fref (middle) and the isolated MBP domain (right) were loaded onto a 4-12% Bis-Tris gel along with a pre-stained molecular weight protein marker (Mr, Proteintech). The gel was run in MES running buffer (NuPAGE, Invitrogen). The gels reveal highly pure preparations of both C2F versions and the MBP control. Arrows indicate the proteins with expected molecular weights. MBP-C2Fwt: 69 kDa; MBP-C2Fref: 69 kDa; MBP single: 43 kDa. **b** Thermal shift assay in the presence of a fluorescence dye to investigate protein stability. Fluorescence-temperature plots normalized to the protein concentration are shown for MBP-C2Fwt (9.4 µM final concentration), MBP-C2Fref (10.8 µM) and MBP (8.7 µM). Buffer was used as a negative control, and its signal was subtracted. mean ± SD are shown for each data point (MBP-C2Fref, *n* = 4; MBP-C2Fwt, *n* = 4; MBP single, *n* = 6). Source data are provided as a Source Data file. **c** First derivative of the fluorescence-temperature plot shown in (b). Note that unfolding of MBP is apparent as a local minimum at ~52 °C in all three constructs. Source data are provided as a Source Data file. **d** Ca^{2+} binding affinity of the wild-type and reframed Dysferlin C2F domains determined by Isothermal Titration Calorimetry at 18 °C. A 150 µM $CaCl_2$ solution was titrated into a solution of MBP-C2Fwt (left, 13.6 µM) or MBP-C2Fref (right, 10 µM). Top panel: Raw heat rates of the binding reactions. Lower panel: Integrated heats and corresponding fit (K_d = (70 ± 30) nM for MBP-C2Fwt and (120 ± 60) nM for MBP-C2Fref).

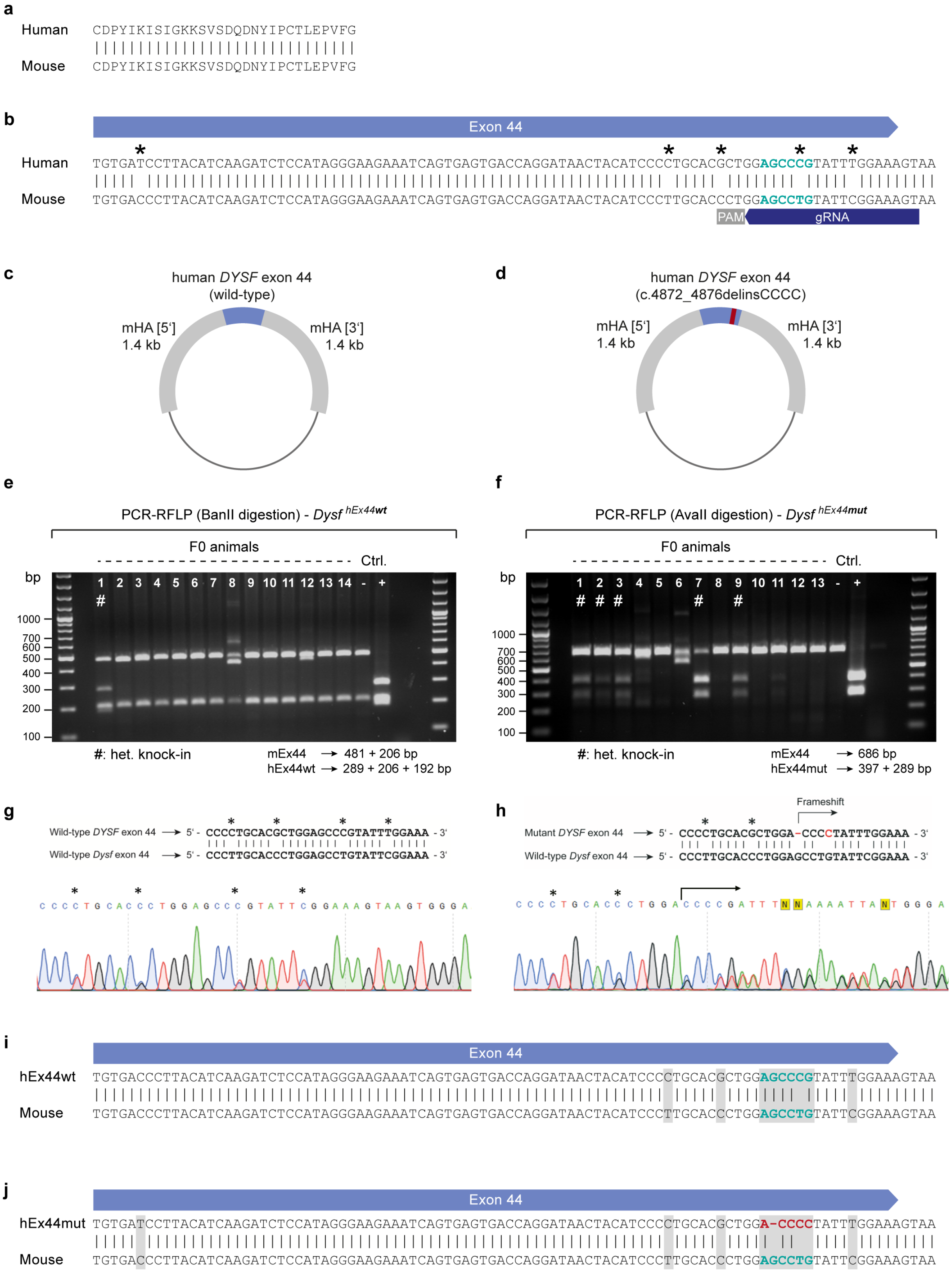

Supplementary Fig. 8: Generation of an LGMD2B mouse model carrying the human *DYSF* exon 44 with the c.4872_4876delinsCCCC founder mutation and a corresponding control. **a** Alignment of the amino acid sequence encoded by the human and murine wild-type *DYSF*/*Dysf* exon 44. **b** Nucleotide sequence alignment of the human and murine wild-type *DYSF*/*Dysf* exon 44. Mismatched

positions are indicated with asterisks. The sequence corresponding to positions c.4872-c.4876 is highlighted in turquoise. The gRNA used to cut on the endogenous mouse exon 44 to generate the transgenic mouse lines is indicated. **c, d** Schematic overview of targeting vectors used to replace the murine exon 44 for the human wild-type (c) or mutant (d) *DYSF* exon 44. mHA: mouse homology arms. **e, f** genotyping strategy of hEx44wt (e) and hEx44mut (f) F0 animals by PCR plus restriction fragment length polymorphism (RFLP). Negative control: Genomic DNA from wild-type C57BL/6N mice. Positive control: Targeting vectors. **g, h** Chromatograms from heterozygous knock-in hEx44wt (g) and hEx44mut (h) F0 animals. **i, j** Nucleotide sequence alignment of the mouse wild-type exon 44 and the hEx44wt (i) and hEx44mut (j) newly generated alleles. The exchanged positions between the mouse genome and the targeting vectors are highlighted with grey squares.

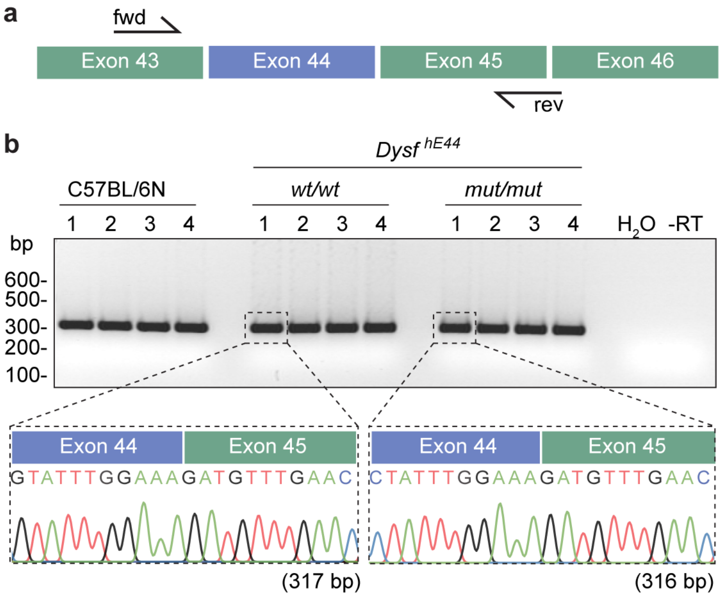

Supplementary Fig. 9: mRNA analysis in homozygous *Dysf* exon 44 humanized mice. a Primer design. **b** RT-PCR analysis of *Dysf* exon 43-46 in homozygous hEx44wt and hEx44mut mice. Wild-type C57BL/6N mice were used as control. The humanized *Dysf* exon 44 (wt or mut) is correctly spliced. The Sanger sequencing chromatograms below show the exon 44-45 junction in homozygous hEx44wt and hEx44mut mice.

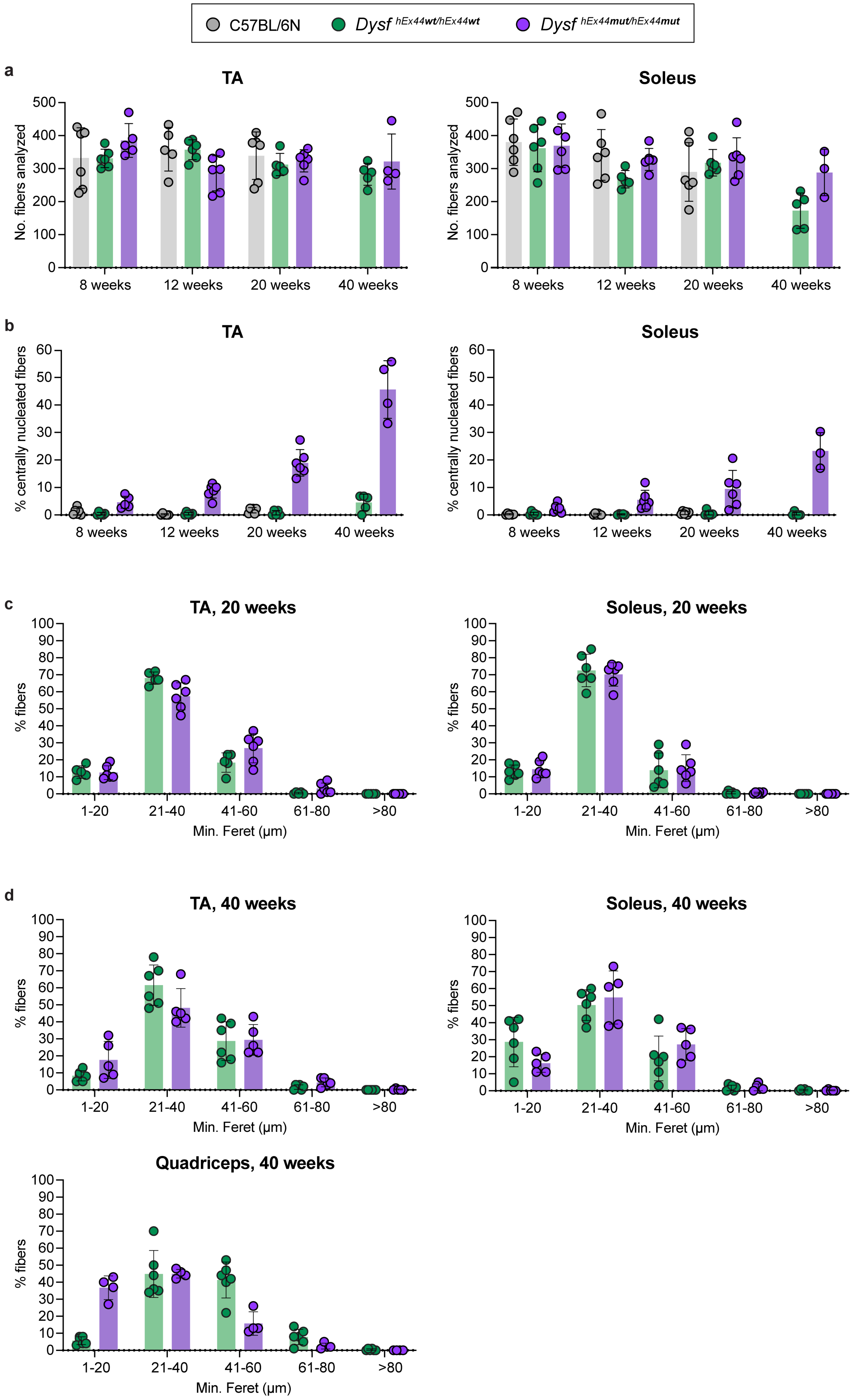
C57BL/6N
Dysf hEx44wt/hEx44wt
Dysf hEx44mut/hEx44mut
a
TA
Soleus
No. fibers analyzed
8 weeks
12 weeks
20 weeks
40 weeks
b
TA
Soleus
% centrally nucleated fibers
c
TA, 20 weeks
Soleus, 20 weeks
% fibers
Min. Feret (μm)
1-20
21-40
41-60
61-80
>80
d
TA, 40 weeks
Soleus, 40 weeks
Quadriceps, 40 weeks

Supplementary Fig. 10: Homozygous hEx44mut mice show a progressive dystrophic phenotype with onset at around 8 weeks of age. a, b Male homozygous hEx44wt and hEx44mut mice were phenotypically analyzed at age 8, 12 and 20 weeks using Gomori's trichrome histological stain and compared to male C57BL/6N wild-type mice. **a** Total number of fibers analyzed for TA (left) and Soleus (right) muscles for each mouse. **b** Percentage of centrally nucleated fibers in TA (left) and Soleus (right) muscles for each mouse. (*n* = 5-6, mean ± SD); *p* values were calculated using a 2-way ANOVA with Tukey's multiple comparisons test. **c** Minimum Feret diameter distribution of TA and Soleus fibers in 20-week-old homozygous hEx44wt and hEx44mut mice. **d** Minimum Feret diameter distribution of TA, Soleus and quadriceps fibers in 40-week-old homozygous hEx44wt and hEx44mut mice. Source data are provided as a Source Data file.

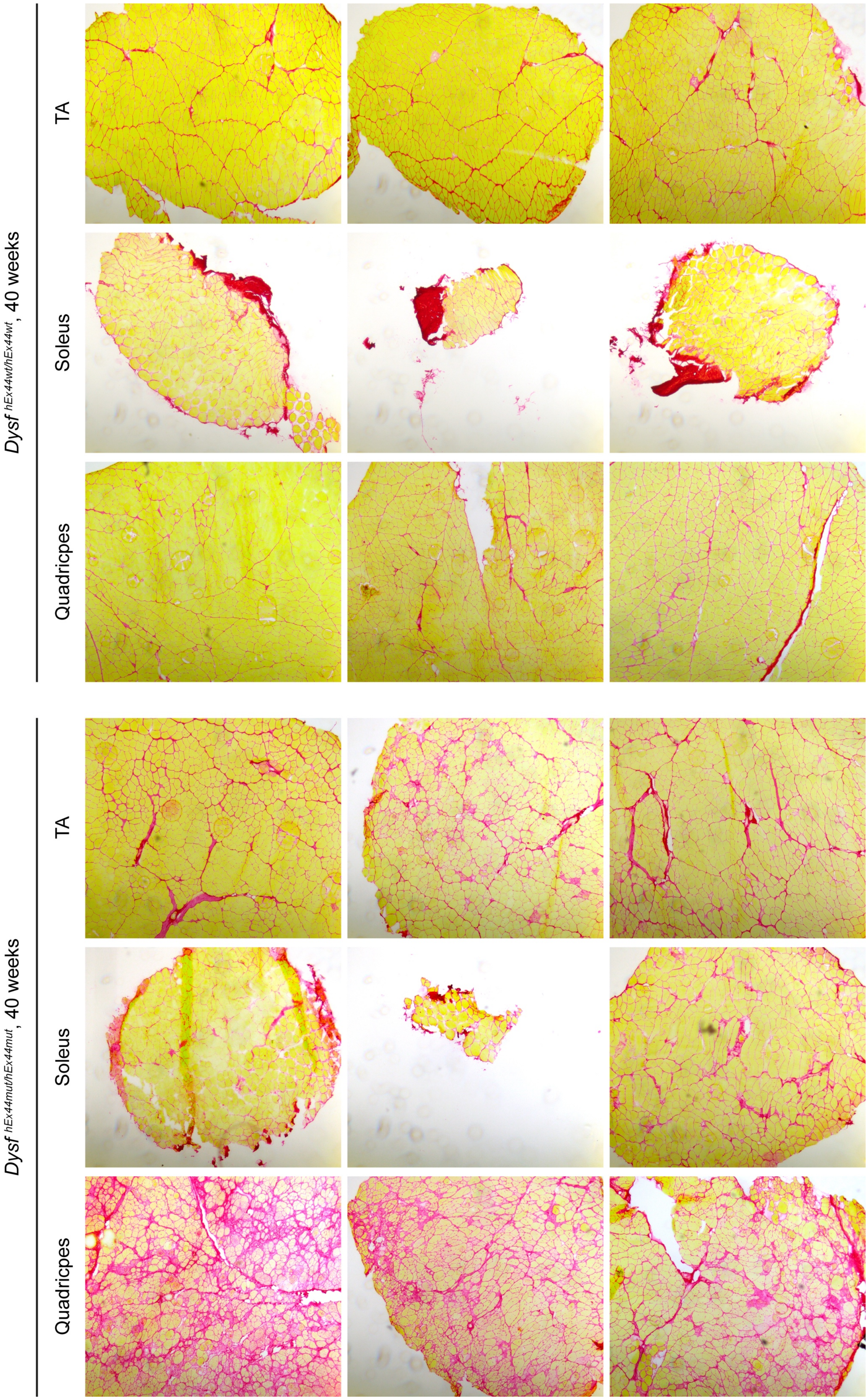

Dysf hEx44wt/hEx44wt, 40 weeks
TA
Soleus
Quadricpes
Dysf hEx44mut/hEx44mut, 40 weeks
TA
Soleus
Quadricpes

Supplementary Fig. 11: Muscle fibrosis is a disease hallmark of homozygous hEx44mut mice. Sirius red staining of 40-week-old homozygous hEx44wt and hEx44mut TA, Soleus and Quadriceps muscles. Low magnification, overview images (*n* = 3 mice per genotype).

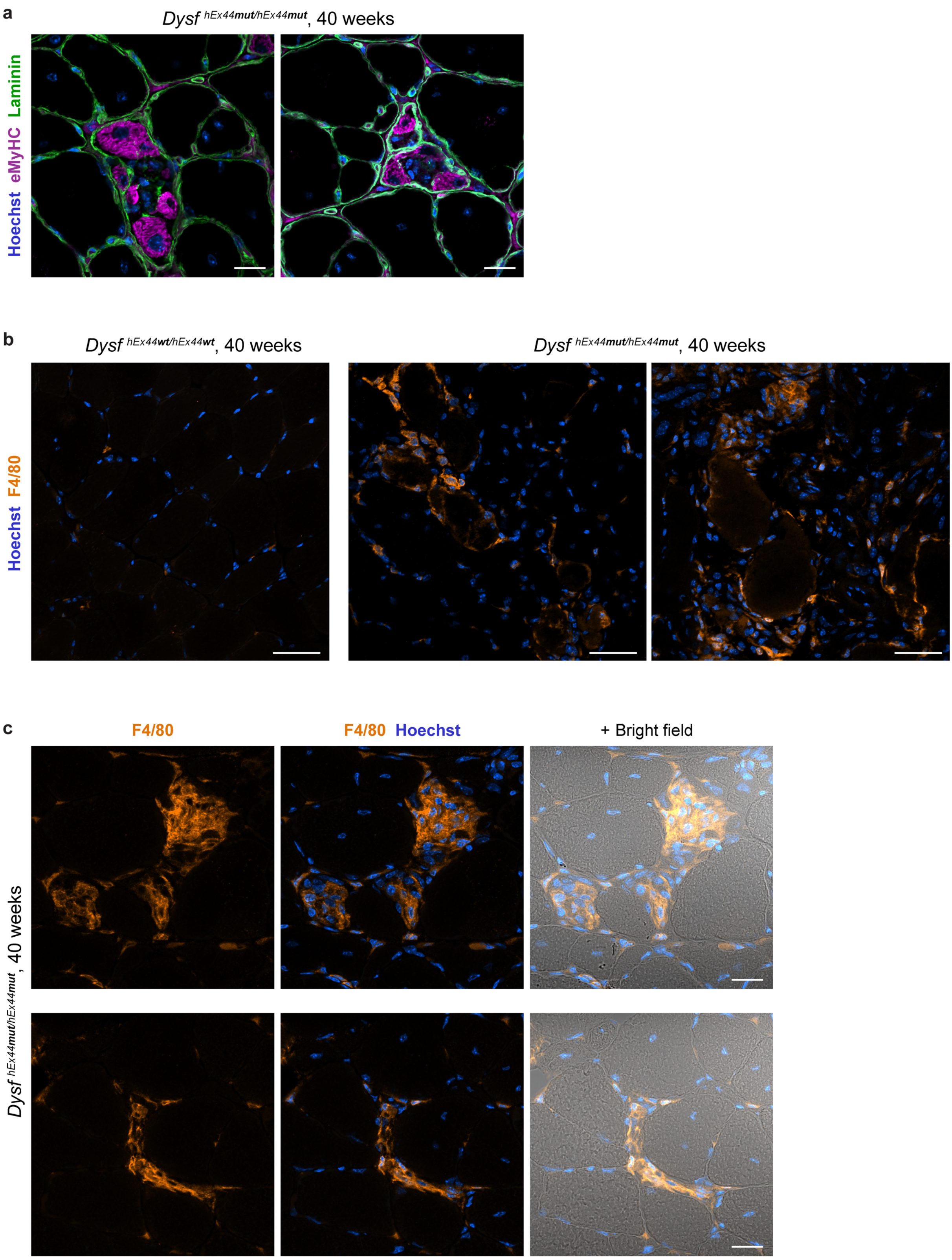

Supplementary Fig. 12: Regenerating fibers and macrophage infiltration are disease hallmarks of homozygous hEx44mut mice. a eMyHC immunostaining of quadriceps cross-sections from 40-week-old homozygous hEx44mut mice shows small clusters of regenerating fibers. Scale bars: 20 µm. **b** F4/80 immunostaining of quadriceps cross-sections from 40-week-old homozygous hEx44wt (left) and hEx44mut (right) mice. Scale bars: 50 µm. **c** Detail images of F4/80-positive macrophage infiltrates in 40-week-old homozygous hEx44mut quadriceps. Scale bars: 20 µm.

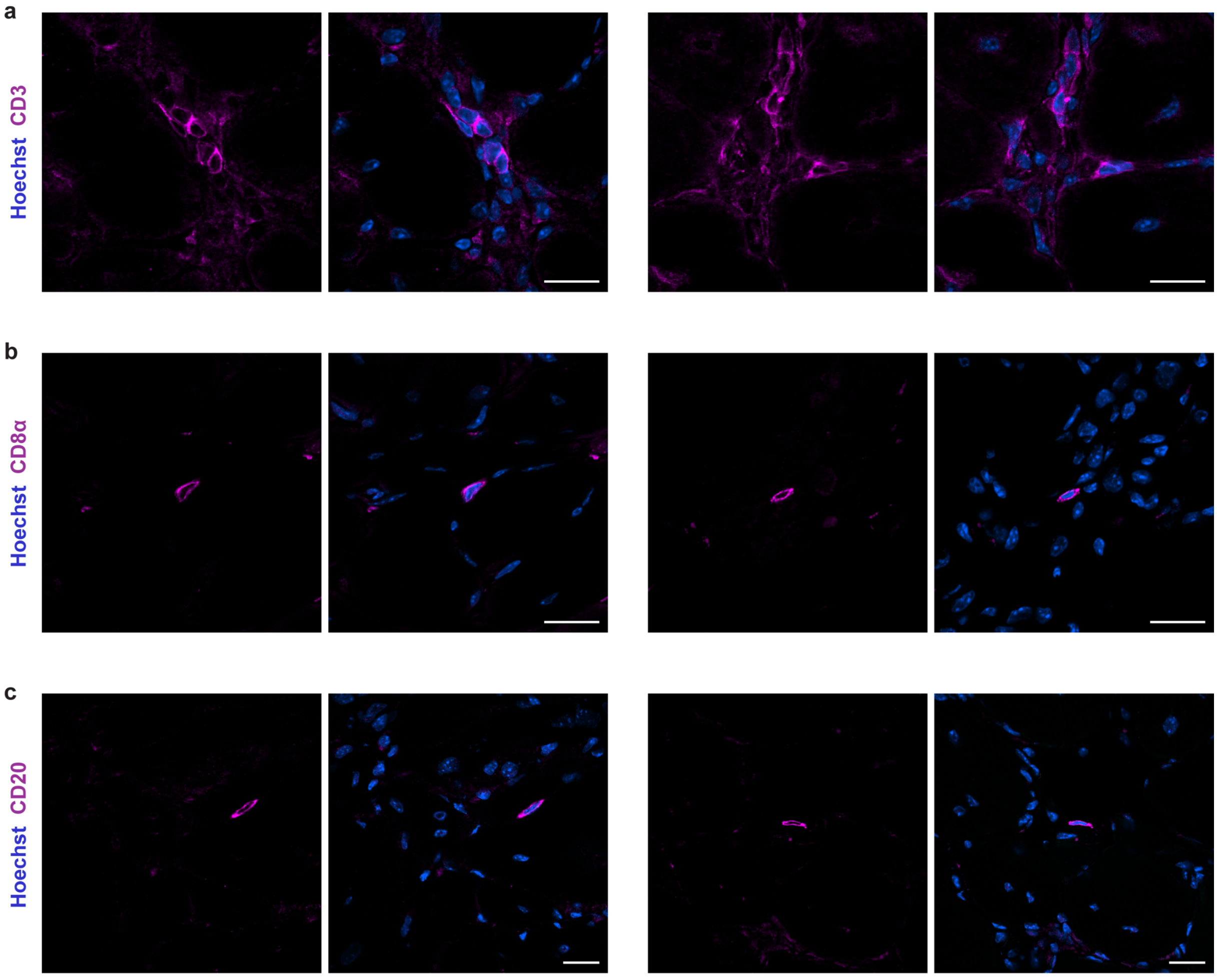

Supplementary Fig. 13: Characterization of lymphocyte infiltrates in homozygous hEx44mut muscles. Quadriceps muscles from 40-week-old homozygous hEx44mut male mice were immunostained for CD3 (**a**) CD8α (**b**) and CD20 (**c**). Scale bars: 20 µm.

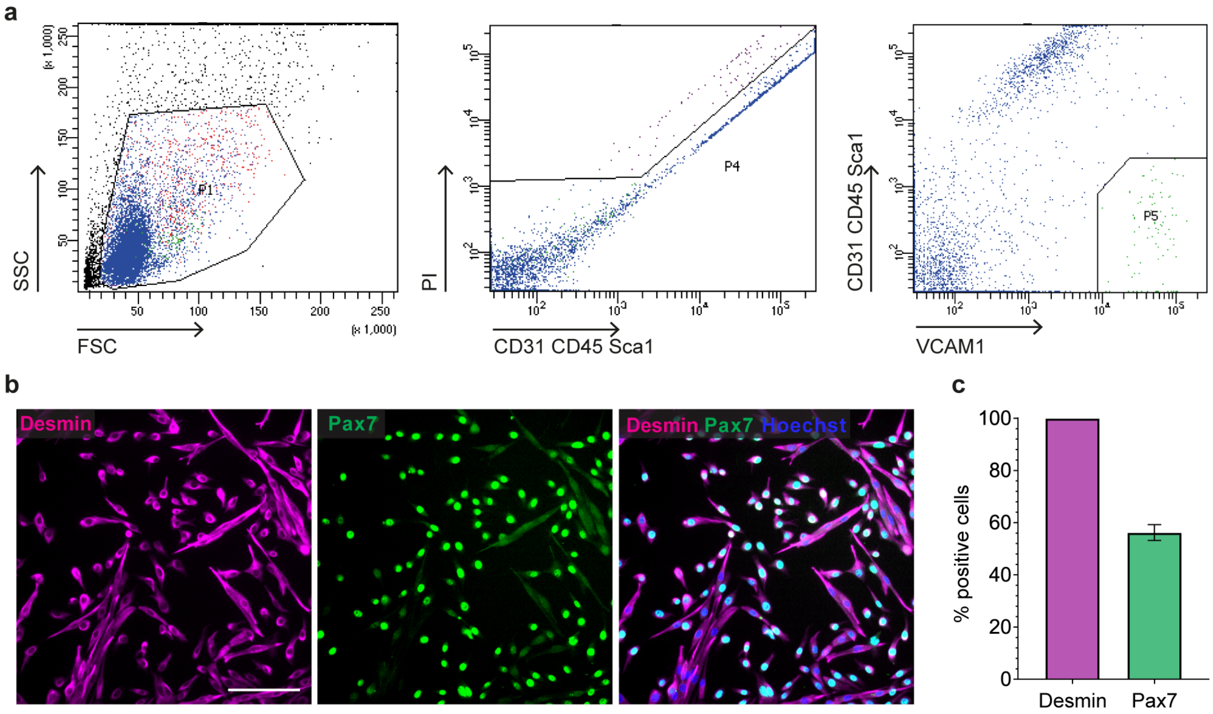

Supplementary Fig. 14: MuSC isolation from *DYSF* exon 44 humanized mice. a Gating strategy. Following tissue digestion and immunostaining, CD31-, CD45-, Sca1- (PE-conjugated primary antibodies) and VCAM1+ cells (Alexa Fluor 488-conjugated secondary antibody) were selected by FACS-sorting (P5 gate). Propidium iodide (PI) was used to exclude dead cells. **b** Immunostaining for myogenic markers Desmin and Pax7 one day after FACS-sorting. Scale bar: 200 µm. **c** Quantification of % cells positive for Desmin and Pax7 one day after FACS-sorting. Source data are provided as a Source Data file.

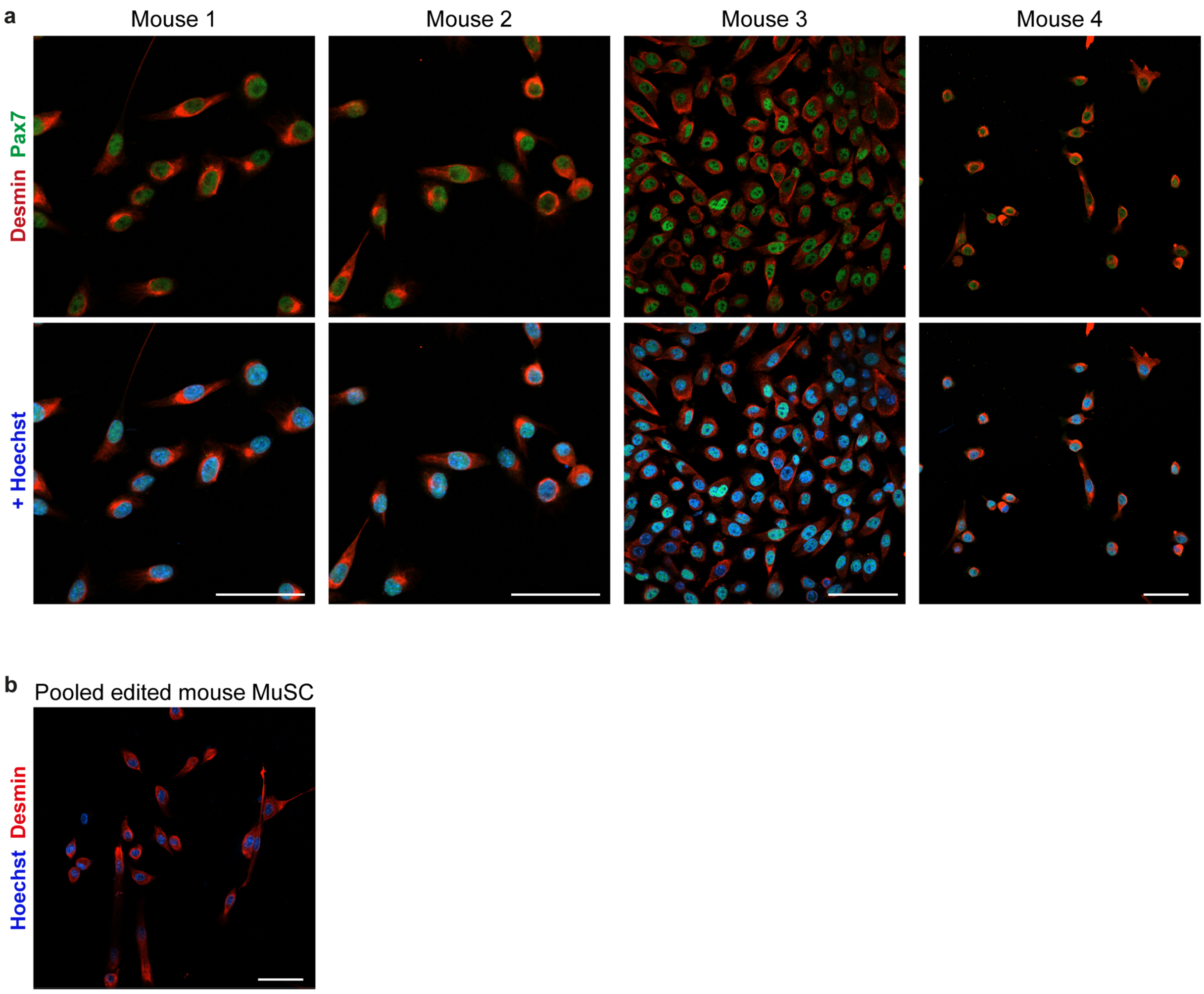

Supplementary Fig. 15: hEx44mut MuSC remained pure and fit following editing. a Pax7/Desmin immunostaining of MuSC from homozygous hEx44mut mice prior to gene editing. Scale bars: 50 µm. **b** MuSC from homozygous hEx44mut mice remained pure after culture and editing, as shown by the Desmin immunostaining of a subset of the gene edited cell populations that were pooled for transplantation. Scale bar: 50 µm.

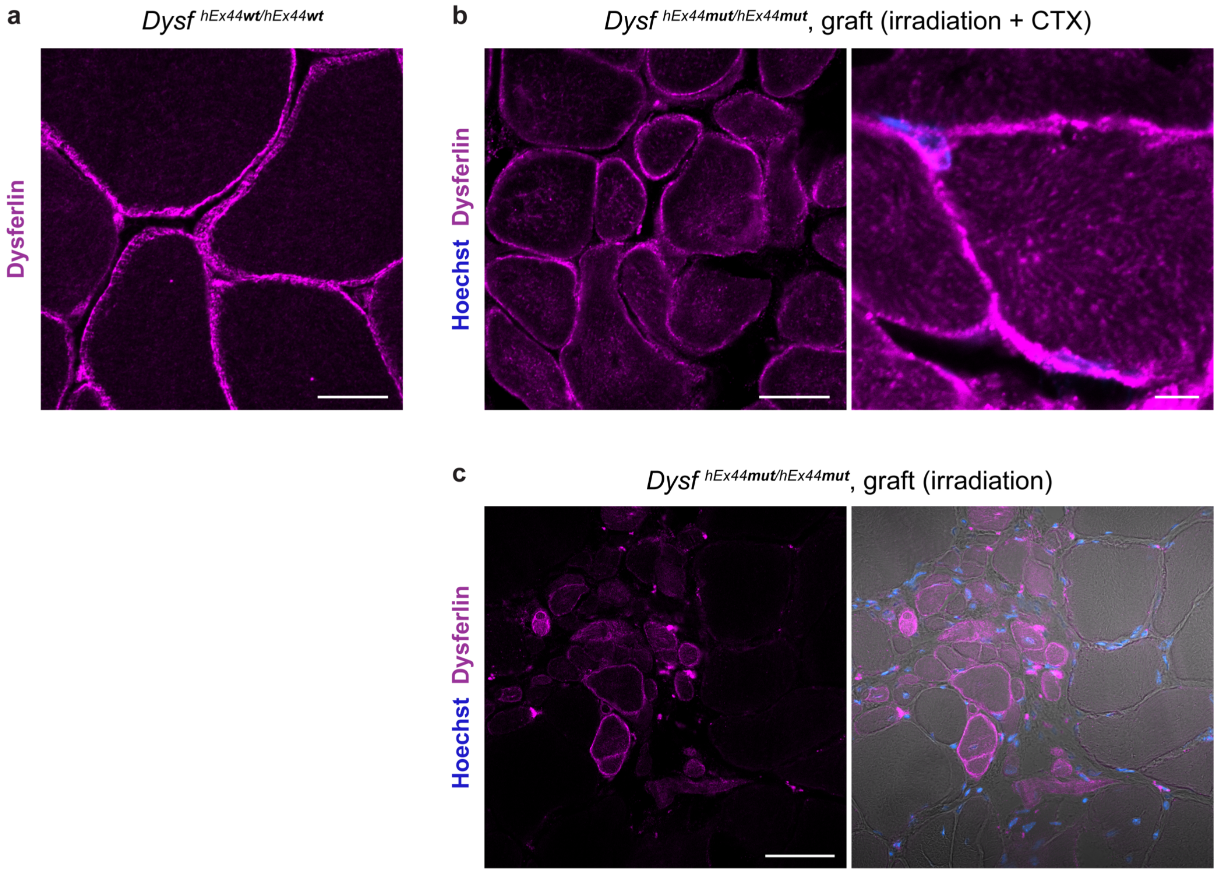

Supplementary Fig. 16: Re-framed dysferlin shows a similar-to-wild-type localization pattern in donor-derived myofibers. Dysferlin immunostaining of transversal cryosections from TA muscles of **a** a homozygous hEx44wt mouse (scale bar: 20 µm), **b** a grafted homozygous hEx44mut mouse pre-treated with irradiation + CTX (left: graft area containing donor-derived, dysferlin-positive fibers, scale bar: 20 µm; right: detail image showing sarcolemmal and reticular localization of reframed dysferlin in donor myofibers, scale bar: 5 µm) and **c** a grafted homozygous hEx44mut mouse pre-treated with irradiation (scale bar: 50 µm).

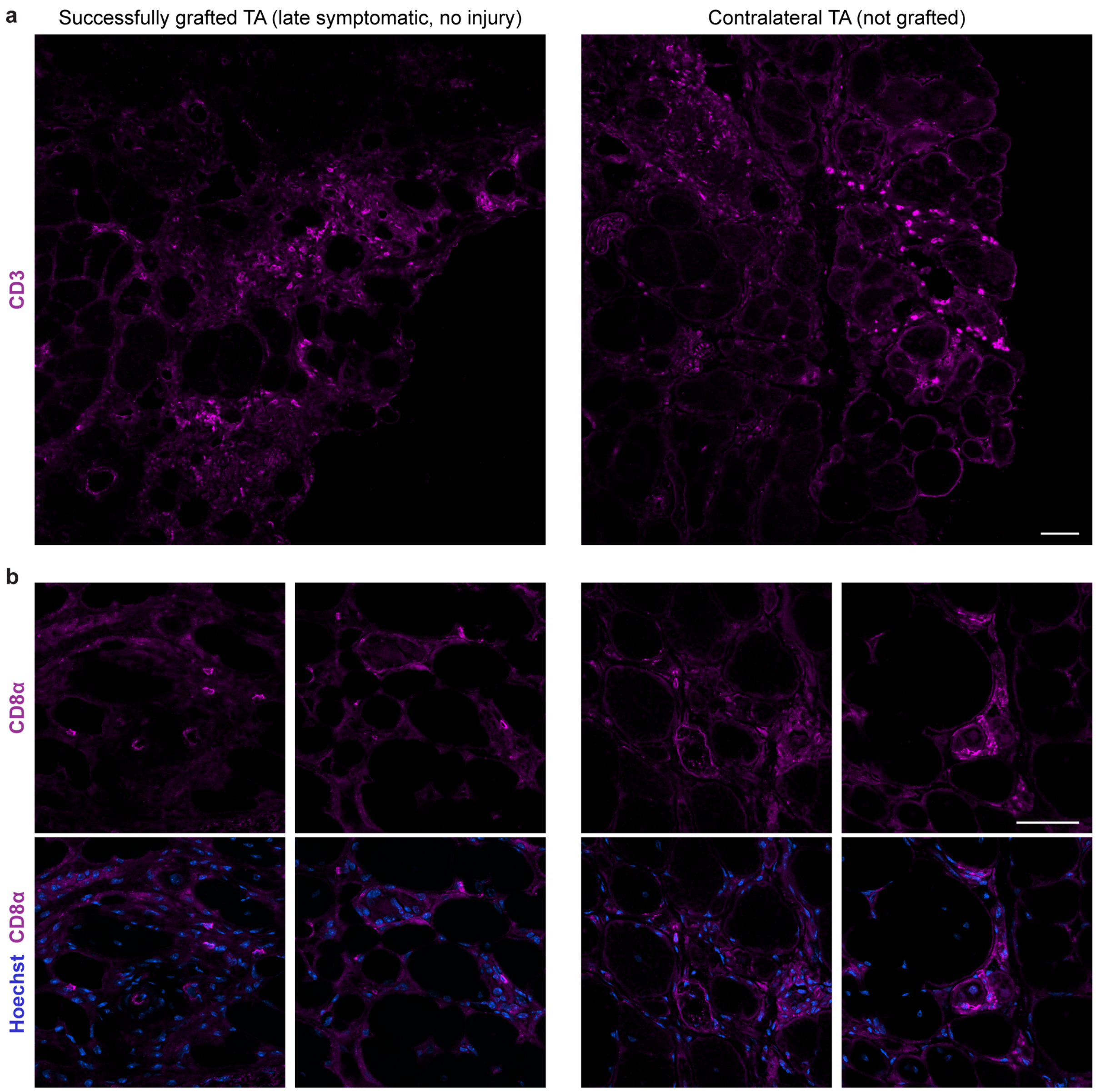

Supplementary Fig. 17: T-Lymphocytes are not apparently enriched in a grafted muscle containing donor-derived fibers. CD3 (**a**) and CD8α (**b**) immunostains on cross-sections from successfully grafted (left) and contralateral, non-grafted (right) TA muscles from a late symptomatic host (no pre-treatment with radiation or CTX). Scale bars: 50 μm.

Supplementary Table 1. MuSC populations used in this study

Age at biopsy (y)	Gender (m/f)	Clinical diagnosis	Muscle histology	MD mutation	Notes
26-30	f	LGMD2B	LGMD2B	*DYSF* exon 44 c.4872-c.4876delinsCCCC	
21-25	f	LGMD2B	LGMD2B	*DYSF* exon 44 c.4872-c.4876delinsCCCC	
	f	Suspected myositis	Normal histology	no	
	f	Suspected myositis	Normal histology	no	
	m	HyperCKemia of unknown cause	Normal histology	no	
46-50	f	Myalgia	Type 2 fiber atrophy	no	GUIDE-seq donor 1
	f	Myalgia	Normal histology	no	GUIDE-seq donor 2
16-20	m	-	Normal histology	no	GUIDE-seq donor 3

Supplementary Table 2. gRNA (spacer) sequences

Name	Sequence 5' > 3'	Target
DYSFex44mut_gRNA#1	CTGCACGCTGGACCCCTATT	*DYSF* exon 44 c.4872delinsCCCC
DYSFex44mut_gRNA#2	CAAATAGGGGTCCAGCGTGC	*DYSF* exon 44 c.4872delinsCCCC
DYSFex44mut_gRNA#3	AAATAGGGGTCCAGCGTGCA	*DYSF* exon 44 c.4872delinsCCCC
-	ACTTTCCGAATACAGGCTCC	Mouse *Dysf* exon 44 (to generate transgenic mice)

Supplementary Table 3. Off-target sites (OTS) analyzed

ID	Locus	OTS sequence (PAM)	Mismatched positions	Chr.	Start	End
**OTS 1**	intergenic:RP11-219A15.4-TNFRSF13B	TCCTTGGGGTCCAGCGTGCAGGG	***.*...............	17	16843496	16843518
**OTS 2**	intron: ARHGEF3	CCATAGAGGTCCAGGTGCAGGG	**....*.......-.....	3	56801671	56801691
**OTS 3**	intergenic:RP11-434D2.7-AC008088.4	TCCTTGGGGTCCAGCGTGCAGGG	***.*...............	17	20515163	20515185
**OTS 4**	intergenic:AL353997.3-LGALS9C	TCCTTGGGGTCCAGCGTGCAGGG	***.*...............	17	18429224	18429246
**OTS 5**	intergenic	TCCTTGGGGTCTAGCGTGCAGGG	***.*......*........	17	30569413	30569434
**OTS 6**	intron:ULK4	AAATAGGGCTCCCAGCATGCACGG	........*.C.....*....	3	41688103	41688125
**OTS 7**	intron:DE4D	AGGAAGGGGTCCAGCTGCAGGG	.***..........-....	5	60249111	60249131
**OTS 8**	intergenic:RP11-468 D11.1-RP11-35O7.1	GAATAGAGGTCCAGCATTCACGG	*.....*........*.*..	5	2849464	2849486
**OTS 9**	exon:SACS	AAAATGGGTTCCAGCCTGCAAGG	...**...*......*....	13	23340392	23340414
**OTS 10**	intergenic:SPHKAP-AC009410.1	AACTTGGAGTCCAGCGTTCAAGG	..*.*..*.........*..	2	228437695	228437717
**OTS 11**	exon:MICALL1	AAATAGGGTCCCAGGGTCCAGGG	........**....*..*..	22	37941104	37941126
**OTS 12**	intergenic:RN7SL795P-GTSCR1	AACTTGGAGTCCAGCGTTCACGG	..*.*..*.........*..	18	70549868	70549890
**OTS 13**	exon:NLRP1/U7	AAATTGGGGTTCAGCGTGGGAGG	....*.....*.......**	17	5514358	5514380
**OTS 14**	exon:NUDT18	GGCCAGGGGTCCAGCGTGCACAG	****................	8	22109233	22109255
**OTS 15**	intron:HMCN2	AAGCAGAGGTGCAGCGTGCAGGG	..**..*...*.........	9	130266781	130266803
**OTS 16**	exon:RP11-580I1.2	CAATAGCAGTCCAGCCTGCATGG	*.....**.......*....	15	24276480	24276502
**OTS 17**	exon:PWRN3	CAATAGCAGTCCAGTGTGCATGG	*.....**......*.....	15	24442761	24442783
**OTS 18**	intron:RP11-305B6.3	AAATAGGGGGAGAACGTGCATGG	.........***.*......	14	44149592	44149614
**OTS 19**	exon:EDF1	AACAAGGGGTCCAGCTTGCGGGG	..**..........*...*	9	136862170	136862192
**OTS 20**	intergenic:CNEP1R1-RP11-429P3.3	AAATGGGACTCCAGCATGCAAGG	....*..**......*....	16	50042948	50042970
**OTS 21**	intron:NR1H4	AAATAGGGGAACAGCATCCAAGG	.........**....*.*..	12	100535509	100535531

Supplementary Table 4. List of oligodeoxynucleotides used in the study

Oligo name	Sequence 5' > 3'	Purpose
HE7/DYSF i43 F	CAGGACACAGCCCACATCT	Human *DYSF* exon 44 PCR
HE8/DYSF i44 R	CTATGCCCCCATAGACATGC	
HE49/Sp_sgRNA_DYSFex44 mut#1_for	CTGCACGCTGGACCCCTATTgtttt	Cloning of spacer sequences into BplI-digested HE_p3.1 (eSpCas91.1) and HE_p4.1 (SpCas9) vector backbones
HE50/Sp_sgRNA_DYSFex44 mut#1_rev	AATAGGGGTCCAGCGTGCAGggtgt	
HE51/Sp_sgRNA_DYSFex44 mut#2_for	CAAATAGGGGTCCAGCGTGCgtttt	
HE52/Sp_sgRNA_DYSFex44 mut#2_rev	GCACGCTGGACCCCTATTTGggtgt	
HE53/Sp_sgRNA_DYSFex44 mut#3_for	AAATAGGGGTCCAGCGTGCAgtttt	
HE54/Sp_sgRNA_DYSFex44 mut#3_rev	TGCACGCTGGACCCCTATTTggtgt	
HE33/humU6prom seq F1	GGCCTATTTCCCATGATTCC	spacer cloning verification (Sanger)
HE85/ssODN-HDR_DYSFex44wt#1	CCATAGGGAAGAAATCAGTGAGTG ACCAGGATAACTACATCCCCTGCAC GCTGGAGCCCGTATTTGGAAAGTA AATTGGGGCATCTTGGGTCTTGGGG TGGAGGAGCCAGACAGGATAAC	HDR template, human wild-type *DYSF* exon 44
HE307/DYSFex44_OT-ig#1_chr5_Fwd	CTCAGAGCCACAGTGGAAGG	Off-target validation by amplicon sequencing
HE308/DYSFex44_OT-ig#1_chr5_Rev	TGAAGAGCTGCTGTTGGGAG	
HE309/DYSFex44_OT-ig#2_chr16_Fwd	AGTGGATGGATGGGTACAGAC	
HE310/DYSFex44_OT-ig#2_chr16_Rev	GCCCACTAAACCACAATTCCT	
HE311/DYSFex44_OT-intron:NR1H4_Fwd	ACCTAGTGTTTGCCAGCAGA	
HE312/DYSFex44_OT-intron:NR1H4_Rev	AGAAGGGTGTGGTTTGGACA	
HE313/DYSFex44_OT-intron:RP11-305B6.3_Fwd	TGCCCAAAACTCCTCATGCT	
HE314/DYSFex44_OT-intron:RP11-305B6.3_Rev	CTGGAGAAACAGGCAGGAGC	
HE315/DYSFex44_OT-ig#3-5_chr17_Fwd	TGGGAGGTTCCTTGGGTACA	
HE316/DYSFex44_OT-ig#3-5_chr17_Rev	CCTGATTCCCATGGCAGGTT	
HE317/DYSFex44_OT-ig#6_chr2_Fwd	AGCTGCCAGGGAATATAAAAC	
HE318/DYSFex44_OT-ig#6_chr2_Rev	GTTGAAGCCTCCAAGTCTGTG	
HE319/DYSFex44_OT-ig#7_chr18_Fwd	TGGGCACCATTTAATCAGTTG	

HE320/DYSFex44_OT-ig#7_chr18_Rev	CCTTGAGTAACAACTGCTAGC	
HE321/DYSFex44_OT-intron:HMCN2_Fwd	CTTGGAGACCGGAATGGCTC	
HE322/DYSFex44_OT-intron:HMCN2_Rev	GACTCTCTACAGTGGAGCGC	
HE323/DYSFex44_OT-exon:NUDT18_Fwd	CGTTGGGTCTTCTGTCCCCA	
HE324/DYSFex44_OT-exon:NUDT18_Rev	CAGCCTATCAGCGGCCAGAG	
HE325/DYSFex44_OT-exon:PWRN3_Fwd	GGCGTCAGTCTTTGTGCAAT	
HE326/DYSFex44_OT-exon:PWRN3_Rev	GGACAGCGATACCTGAGACA	
HE327/DYSFex44_OT-exon:NLRP1/U7_Fwd	CTGTTGGCTTGCTCTTGTAGA	
HE328/DYSFex44_OT-exon:NLRP1/U7_Rev	GCCTTCCAGCACTAAAGTAATG	
HE329/DYSFex44_OT-exon:MICALL1_Fwd	TGCTCCCCTCAGATCAGTCA	
HE330/DYSFex44_OT-exon:MICALL1_Rev	CTGGAAGAGCAGAACCCTGG	
HE331/DYSFex44_OT-exon:SACS_Fwd	TTCCAGACCAAAGAGCCTGG	
HE332/DYSFex44_OT-exon:SACS_Rev	GCTGGTGAACTCTTTGACCCT	
HE333/DYSFex44_OT-exon:RP11-580I1.2_Fwd	GGTATGTGTCCACTTGGTTGG	
HE334/DYSFex44_OT-exon:RP11-580I1.2_Rev	GACTGGCTGGATCGCATCTA	
HE335/DYSFex44_OT-exon:EDF1_Fwd	GAGGAACGCGATGTAGGGAG	
HE336/DYSFex44_OT-exon:EDF1_Rev	AGATGTTGAAGAGGGGCAGC	
StM1/DYSFex44_OT-chr5_1_fwd	GCCACCACTGTTTCCTTCTG	
StM2/DYSFex44_OT-chr5_1_rev	GAATGATCCATTTCACTAAG	
StM3/DYSFex44_OT-chr3_1_fwd	GACTGTGGGAGAGATGTAGAG	
StM4/DYSFex44_OT-chr3_1_rev	GGCTGTCGGTCTCCGTGTC	
StM5/DYSFex44_OT-chr3_2_fwd	GGGAGGTGGACGCAAACATG	
StM6/ DYSFex44_OT-chr3_2_rev	GATGGGGGTTCCACTATGTG	
StM7/DYSFex44_OT-chr17_1_fwd	CAAGATTTCCTTACTTATCCC	
StM8/DYSFex44_OT-chr17_1_rev	GAGCTGACCCTGGCCAGAGC	

StM9/DYSFex44_OT-chr17_2_fwd	GTTTGAAATGGGACAAGAAGG	
StM10/DYSFex44_OT-chr17_2_rev	GCTGGATGGGCTGACCTTG	
HE14/pMX seq R	CCCAATACGCAAGGAAACAG	Sequencing of mTV_hDYSF_ex44 wt&mut inserts from GeneArt pMX vector
HE15/mDysf i43 F2	GTTGGAAAGGGAGGGAGAAC	
HE16/mDysf i44 R2	TGAAAAGGTCTCTTGGGAAGG	
HE17/mDysf i44 F	CCACCAGTTTCTCTTTCTGTCC	
HE12/Dysf i43 F	GCTTTCCTTGTGTGCCAGTG	Mouse *Dysf* exon 44 (& hEx44) PCR-RFLP genotyping and genome editing analysis by Sanger
HE13/Dysf i44 R	TAAGGGGAAGGTGGGGTGAA	
HE223/mDysf e43 F	CAGGAATGCTTGGTCCGTAT	PCR plus Sanger sequencing of the complete hEx44mut and hEx44wt knock-in alleles
HE224/mDysf i43 F2	TTTGGGCTTCTGGAAAAATG	
HE225/mDysf e45 F	AGAAGATTGGGGAGACGGTC	
HE226/mDysf e45 R	GACCGTCTCCCCAATCTTCT	
HE337/mDysf i43 F2	AATGGGTGAACGGGTGAGAT	Mouse *Dysf* exon 44 (& hEx44) PCR and genome editing analysis by NGS
HE338/mDysf i44 R2	CCCACCCAAGGAATTAGAGACT	
SDFp_32	ATCGTCCGAGCATTTGGCTTA	Mouse *Dysf* exon 43-46, RT-qPCR
SDFp_33	GGTCCAGAGACACAGTAGGTC	
oAK26/Gapdh_ex4 F1	GCATCCTGCACCACCAACTG	Mouse *Gapdh*, RT-qPCR
oAK27/Gapdh_ex5 R1	CATCACGCCACAGCTTTCCA	
I5_Nextera_3’_GSP-	TCGTCGGCAGCGTCAGATGTGTATAAGAGACAGGTTTAATTGAGTTGTCATATGTTAATAACGGT	GUIDE-seq
I5_Nextera_5’_GSP+	TCGTCGGCAGCGTCAGATGTGTATAAGAGACAGATACCGTTATTAACATATGACAACTCAATTAA	
I7_Nextera_R1	GTCTCGTGGGCTCGGAGATGTGTATAAG	
N7XX index (i7)	CAAGCAGAAGACGGCATACGAGATXXXXXXXXGTCTCGTGGGCTCGG	
S5XX index (i5)	AATGATACGGCGACCACCGAGATCTACACXXXXXXXXTCGTCGGCAGCGTC	
JS/C2F_1575-1792_fwd	CCCCAGGAGTGCTTGGTC	Cloning of *DYSF* C2F-domain expression plasmids
JS/C2F_1575-1792_rev	CTTCGGAAATAGGTCGACCC	

Supplementary Table 5. Short tandem repeat (STR) analysis of patient hiPSC and blood

	Patient 1, blood	Patient 1, hiPSC	Patient 2, blood	Patient 2, hiPSC
D10S1248	13	13	13	13
Vwa	14,16	14,16	14,16	14,16
D16S539	11,14	11,14	13,14	13,14
D2S1338	17,23	17,23	17,18	17,18
D8S1179	10,11	10,11	10,11	10,11
D21S11	27,30	27,30	28,31	28,31
D19S51	13,16	13,16	12,13	12,13
D22S1045	15,16	15,16	15,17	15,17
D19S433	14	14	14	14
TH01	6,7	6,7	6,7	6,7
FGA	21,23	21,23	21,23	21,23
D2S441	14	14	14	14
D3S1358	14	14	17,18	17,18
D1S1656	12,15*	12,16*	12,16	12,16
D12S391	16,22	16,22	16,22	16,22
SE33	17,25.2	17,25.2	18,25.2	18,25.2
Amelogenin	X	X	X	X

*likely tissue-specific mutation

Supplementary Table 6. Primary antibodies

Antibody	Clone	Manufacturer & catalogue number	Working dilution
PAX7		Santa Cruz Biotechnology, # sc-81648	IF - 1:300
PAX7	P3U1	Developmental studies hybridoma bank (DSHB). Produced in-house from hybridoma cell line.	IF tissue - undiluted cell culture supernatant
Ki-67		Thermo Fisher Scientifitc, # RM-9106-S0	IF - 1:300
MYF5	C20	Santa Cruz Biotechnology, # sc-302	IF - 1:2,000
MYOD	5.8A	Santa Cruz Biotechnology, # sc-32758	IF - 1:50
Desmin		Dako, # M0760	IF -1:100
Desmin		Abcam, # ab15200	IF - 1:2,000
Skeletal Myosin (fast)	MY-32	Sigma-Aldrich, # M4276	IF - 1:500
Dysferlin (Hamlet)		Novocastra, # NCL-Hamlet	WB - 1:500
Dysferlin (Romeo)		Abcam, # ab124684	IF & WB - 1:150<br>IF tissue - 1:50
Annexin A1		Abcam, # ab88865	IF - 1:80
α-tubulin		Sigma-Aldrich, # T5168	WB - 1:2,000
Vinculin	VIN-11-5	Sigma-Aldrich, # V4505	WB - 1:200
VCAM1		R&D systems, # AF643	FACS - 1:100
PE-anti CD31	MEC13.3	DB Pharmingen, # 553373	FACS - 1: 200
PE-anti CD45	30F11	DB Pharmingen, # 553081	FACS - 1: 200
PE-anti Sca1	E13-161.7	DB Pharmingen, # 553336	FACS - 1: 200
Laminin		Sigma-Aldrich, # L9393	IF tissue - 1:200
eMyHC	F1.652	DSBH, # F1.652	IF tissue - undiluted cell culture supernatant
F4/80	A3-1	Invitrogen, # MA1-91124	IF tissue – 1:1,000
Mouse CD3	17A4	R&D Systems, # MAB4841	IF tissue – 1:100
Mouse CD8α	53-6.7	R&D Systems, # MAB116	IF tissue – 1:100
CD20	SP32	Abcam, # ab64088	IF tissue – 1:100

IF: Immunofluorescence staining

WB: Western blot
