# Supplementary material for: Gene-editing in patient and humanized-mice primary muscle stem cells rescues dysferlin expression in dysferlin-deficient muscular dystrophy
Source: Nat Commun. 2025 Jan 2;16:120. doi: 10.1038/s41467-024-55086-0 (PMC11695731; doi:10.1038/s41467-024-55086-0)
Supplement: Supplementary file 2 — Reporting Summary [file 41467_2024_55086_MOESM2_ESM.pdf]

nature portfolio

Corresponding author(s): Helena Escobar, Simone Spuler

Last updated by author(s): Aug 14, 2024

Reporting Summary

Nature Portfolio wishes to improve the reproducibility of the work that we publish. This form provides structure for consistency and transparency in reporting. For further information on Nature Portfolio policies, see our Editorial Policies and the Editorial Policy Checklist.

Statistics

For all statistical analyses, confirm that the following items are present in the figure legend, table legend, main text, or Methods section.

n/a	Confirmed	
☐	☒	The exact sample size (*n*) for each experimental group/condition, given as a discrete number and unit of measurement
☒	☐	A statement on whether measurements were taken from distinct samples or whether the same sample was measured repeatedly
☐	☒	The statistical test(s) used AND whether they are one- or two-sided<br>*Only common tests should be described solely by name; describe more complex techniques in the Methods section.*
☒	☐	A description of all covariates tested
☒	☐	A description of any assumptions or corrections, such as tests of normality and adjustment for multiple comparisons
☐	☒	A full description of the statistical parameters including central tendency (e.g. means) or other basic estimates (e.g. regression coefficient) AND variation (e.g. standard deviation) or associated estimates of uncertainty (e.g. confidence intervals)
☐	☒	For null hypothesis testing, the test statistic (e.g. *F*, *t*, *r*) with confidence intervals, effect sizes, degrees of freedom and *P* value noted<br>*Give P values as exact values whenever suitable.*
☒	☐	For Bayesian analysis, information on the choice of priors and Markov chain Monte Carlo settings
☒	☐	For hierarchical and complex designs, identification of the appropriate level for tests and full reporting of outcomes
☒	☐	Estimates of effect sizes (e.g. Cohen's *d*, Pearson's *r*), indicating how they were calculated

Our web collection on statistics for biologists contains articles on many of the points above.

Software and code

Policy information about availability of computer code

Data collection	CRISPOR (online tool: http://crispor.gi.ucsc.edu), ZEN desk (Carl Zeiss Microscopy), LAS X (Leica Microsystems), CrispRGold v.1.2 (doi: 10.1073/pnas.1613884113), VWR® Image Capture Software (VWR), CFX Maestro (Bio-Rad), AlphaFold2 (doi: 10.1038/s41586-021-03819-2), Coot (doi: 10.1107/S0907444910007493), Origin® (MicroCal™, GE Healthcare)
Data analysis	ICE v2.0 (Synthego), CRISPResso2 (http://crispresso2.pinellolab.org), ZEN 3.4 Blue edition (Carl Zeiss Microscopy), ImageJ/Fiji (NIH), Myosoft (doi: 10.1371/journal.pone.0229041), PyMOL 2.3.2 (Schrödinger), Origin® (MicroCal™, GE Healthcare), Adobe Photoshop CC 17, Adobe Illustrator (2023), Microsoft Excel, GraphPad Prism

For manuscripts utilizing custom algorithms or software that are central to the research but not yet described in published literature, software must be made available to editors and reviewers. We strongly encourage code deposition in a community repository (e.g. GitHub). See the Nature Portfolio guidelines for submitting code & software for further information.

Data

Policy information about availability of data

All manuscripts must include a data availability statement. This statement should provide the following information, where applicable:

- Accession codes, unique identifiers, or web links for publicly available datasets
- A description of any restrictions on data availability
- For clinical datasets or third party data, please ensure that the statement adheres to our policy

Raw NGS data are available at the NCBI Sequence Read Archive (SRA) under the Bioproject accession no... (will be available before publication). All research data

supporting the claims of the study is available upon reasonable request to the corresponding authors.

Research involving human participants, their data, or biological material

Policy information about studies with human participants or human data. See also policy information about sex, gender (identity/presentation), and sexual orientation and race, ethnicity and racism.

Reporting on sex and gender	Details on the muscle stem cell donors included in the study are provided in Supplementary Table 1. Eight donors were included (six females and two males). Dysferlin-deficient muscular dystrophy (LGMD2B/R2) affects both sexes but only two female patients carrying the relevant mutation were available as muscle biopsy donors.
Reporting on race, ethnicity, or other socially relevant groupings	Race and ethnicity were not considered due to the small group size and the limitation of available samples/donors.
Population characteristics	Details on the muscle stem cell donors included in the study are provided in Supplementary Table 1. Eight donors were included (age 16-50, six women and two men). Two had a genetic diagnosis of LGMD2B. Six had a normal muscle histology and/or no genetic diagnosis and were considered controls in this study.
Recruitment	All donors were seen at the Outpatient Clinic for Muscle Disorders of the Charité - Universitätsmedizin Berlin.
Ethics oversight	The regulatory agencies (EA2/051/10 and EA2/175/17, Charité – Universitätsmedizin Berlin) approved the studies and written informed consent was obtained from donors or legal guardians.

Note that full information on the approval of the study protocol must also be provided in the manuscript.

Field-specific reporting

Please select the one below that is the best fit for your research. If you are not sure, read the appropriate sections before making your selection.

[x] Life sciences [ ] Behavioural & social sciences [ ] Ecological, evolutionary & environmental sciences

For a reference copy of the document with all sections, see nature.com/documents/nr-reporting-summary-flat.pdf

Life sciences study design

All studies must disclose on these points even when the disclosure is negative.

Sample size	Sample size was determined based on previous experience and with at least n = 3 independent experiments/human donors/mice whenever possible, or at least n = 3 technical repeats per experiment/human donor/mouse when independent experiments/human donors/mice were not possible. For the phenotyping of the newly generated mouse models, n = 6 was used whenever possible. Sample size is indicated in the manuscript for all experiments.
Data exclusions	No data were excluded from the analysis.
Replication	Gene editing experiments and their functional outcome, as well as other experiments in the manuscript, including the human and mouse iPSC and muscle stem cell characterization, analysis of the grafts and phenotyping of the novel mouse models, have been reproduced by several scientists in the Spuler laboratory. Data has been shared and discussed with lab members and relevant collaborators.
Randomization	Random allocation of human samples samples was not possible due to the small sample size. Samples were defined as either patient or control, and treatments were applied to both whenever possible. For the phenotyping of the newly generated mouse models, mice of the appropriate genotype, age and sex were selected randomly from the available colony whenever possible. For experiments involving mouse muscle stem cell isolation and transplantation, mice were selected based on genotype, sex, age and availability of siblings as donors/recipients for muscle stem cell grafts where applicable.
Blinding	Blinding was applied to the laser injury experiment and to the histological analysis performed as part of the phenotyping of the new mouse models. In the rest of the experiments blinding was not performed because either data analaysis and interpretation cannot be subjective (e.g. genome editing/sequencing /RT-qPCR data) or differences between samples/conditions were obvious at first glance (e.g. Western blots - where, in addition, samples were loaded into the gel pockets following a defined sequence). In the analysis of the grafts, blinding could not be applied but the contralateral non-transplanted muscle was sectioned and stained in parallel as an internal control whenever available (e.g. for all mice in the old symptomatic, non-pre-injured cohort).

Reporting for specific materials, systems and methods

We require information from authors about some types of materials, experimental systems and methods used in many studies. Here, indicate whether each material, system or method listed is relevant to your study. If you are not sure if a list item applies to your research, read the appropriate section before selecting a response.

Materials & experimental systems

n/a	Involved in the study
☐	☒ Antibodies
☐	☒ Eukaryotic cell lines
☒	☐ Palaeontology and archaeology
☐	☒ Animals and other organisms
☒	☐ Clinical data
☒	☐ Dual use research of concern
☒	☐ Plants

Methods

n/a	Involved in the study
☒	☐ ChIP-seq
☐	☒ Flow cytometry
☒	☐ MRI-based neuroimaging

Antibodies

Antibodies used	All primary antibodies with corresponding working concentrations/dilutions are listed in Supplementary Table 6:<br>PAX7: Clone P3U1. Provider: Santa Cruz Biotechnology, # sc-81648 / Developmental studies hybridoma bank (DSHB) and in-house produced undiluted cell culture supernatant.<br>Ki-67: Thermo Fisher Scientifitc, # RM-9106-S0.<br>MYF5: Clone C20. Santa Cruz Biotechnology, # sc-302.<br>MYOD: Clone 5.8A. Santa Cruz Biotechnology, # sc-32758.<br>Desmin: Dako, # M0760; Abcam, # ab15200.<br>Skeletal Myosin (fast): Clone MY-32. Sigma-Aldrich, # M4276.<br>Dysferlin: Novocastra, # NCL-Hamlet; Abcam, # ab124684.<br>Annexin A1: Abcam, # ab88865.<br>α-tubulin: Sigma-Aldrich, # T5168<br>Vinculin: Clone VIN-11-5. Sigma-Aldrich, # V4505.<br>VCAM1: R&D systems, # AF643.<br>PE-anti CD31: Clone MEC13.3. DB Pharmingen, # 553373.<br>PE-anti CD45: Clone 30F11. DB Pharmingen, # 553081.<br>PE-anti Sca1: Clone E13-161.7. DB Pharmingen, # 553336.<br>Laminin: Sigma-Aldrich, # L9393.<br>eMyHC: Clone F1.652. DSBH, # F1.652.<br>F4/80: Clone A3-1. Invitrogen, # MA1-91124.<br>Mouse CD3: Clone 17A4. R&D Systems, # MAB4841.<br>Mouse CD8α: Clone 53-6.7. R&D Systems, # MAB116.<br>CD20: Clone SP32. Abcam, # ab64088.
Validation	All antibodies used in the study have been extensively validated by the authors for the methods in which they have been used. Whenever possible, absence of signal from a primary antibody in a negative control consisting of a knock-out of the corresponding protein, or cells/tissues that do not express the protein, has been used as validation criteria.

Eukaryotic cell lines

Policy information about cell lines and Sex and Gender in Research

Cell line source(s)	Human induced pluripotent stem cell (hiPSC) lines used in the study were generated in the Spuler laboratory and are described in the study or published (the corresponding references are included).
Authentication	All hiPSC lines were authenticated by Short tandem repeat (STR) analysis and compared to blood from the corresponding donors.
Mycoplasma contamination	All cell lines used were tested negative for mycoplasma contamination.
Commonly misidentified lines<br>(See ICLAC register)	N.A.

Animals and other research organisms

Policy information about studies involving animals; ARRIVE guidelines recommended for reporting animal research, and Sex and Gender in Research

Laboratory animals	Mice (Mus musculus)
Wild animals	N.A.
Reporting on sex	Details on the sex of the animals used are included in the methods. Only male mice were included in the study (except for MuSC donors, where both males and female mice were used). Although the phenotypic characterization of the newly generated mouse models is shown only for male mice, most of the histological characterization was also performed for age-matched female mice with

similar findings.

Field-collected samples: N.A.

Ethics oversight: Animal experiments were performed under the license numbers G0162/12, G0111/17, G0301/18 and G0223/20 (Landesamt für Gesundheit und Soziales – LaGeSo – Berlin, Germany).

Note that full information on the approval of the study protocol must also be provided in the manuscript.

Plants

Seed stocks: *Report on the source of all seed stocks or other plant material used. If applicable, state the seed stock centre and catalogue number. If plant specimens were collected from the field, describe the collection location, date and sampling procedures.*

Novel plant genotypes: *Describe the methods by which all novel plant genotypes were produced. This includes those generated by transgenic approaches, gene editing, chemical/radiation-based mutagenesis and hybridization. For transgenic lines, describe the transformation method, the number of independent lines analyzed and the generation upon which experiments were performed. For gene-edited lines, describe the editor used, the endogenous sequence targeted for editing, the targeting guide RNA sequence (if applicable) and how the editor was applied.*

Authentication: *Describe any authentication procedures for each seed stock used or novel genotype generated. Describe any experiments used to assess the effect of a mutation and, where applicable, how potential secondary effects (e.g. second site T-DNA insertions, mosiacism, off-target gene editing) were examined.*

Flow Cytometry

Plots

Confirm that:

- [x] The axis labels state the marker and fluorochrome used (e.g. CD4-FITC).
- [x] The axis scales are clearly visible. Include numbers along axes only for bottom left plot of group (a 'group' is an analysis of identical markers).
- [x] All plots are contour plots with outliers or pseudocolor plots.
- [x] A numerical value for number of cells or percentage (with statistics) is provided.

Methodology

Sample preparation: Mouse muscle stem cells were isolated from mouse hind limbs as described (Bröhl et al., 2012 Dev Cell). Briefly, muscle tissue was dissected, mechanically minced, and digested with NB4 collagenase (12mg/ml) (SERVA) and Dispase II (100U/ml) (Roche) for 1 hour, and TrypLE Express (Gibco) for 5 minutes. The resulting cell suspension was stained with PE-conjugated anti-Sca1, -CD31 and -CD45 antibodies and an anti-VCAM1 primary antibody (Supplementary Table 6) plus a secondary Alexa Fluor 488-conjugated secondary antibody. Sca1(neg), CD31(neg), CD45(neg), VCAM1(pos) cells were selected.

Instrument: FACSAria Cell Sorter (BD Biosciences)

Software: FlowJo

Cell population abundance: Pax7/Desmin immunostaining was performed on the post-sort fraction to determine purity.

Gating strategy:
SSC-A/FSC-A > P1
FSC-W/FSC-A > P2
SSC-W/SSC-A > P3
PI/PE(CD31,C45,Sca1) > P4
PE/A488(VCAM1) > P5

The P5 (VCAM1+, CD31-, CD45-, Sca1-) fraction was collected.

Negative controls for all single stainings were used to setp up the gates. Gates were stringent with no overlap between the positive and negative populations.

- [x] Tick this box to confirm that a figure exemplifying the gating strategy is provided in the Supplementary Information.
